# A prospective observational study on critically ill children with diaphragmatic dysfunction: clinical outcomes and risk factors

**DOI:** 10.1186/s12887-020-02310-7

**Published:** 2020-09-04

**Authors:** Yang Xue, Chun-Feng Yang, Yu Ao, Ji Qi, Fei-Yong Jia

**Affiliations:** 1 Department of Developmental and Behavioral Pediatrics, The First Hospital of Jilin University, 71 Xinmin Street, Changchun, 130021 China; 2 Department of Pediatrics Intensive Care Unit, The First Hospital of Jilin University, Changchun, China

**Keywords:** Diaphragm, Mechanical ventilation, Children, Ultrasound

## Abstract

**Background:**

Diaphragmatic dysfunction (**DD**) has a great negative impact on clinical outcomes, and it is a well-recognized complication in adult patients with critical illness. However, DD is largely unexplored in the critically ill pediatric population. The aim of this study was to identify risk factors associated with DD, and to investigate the effects of DD on clinical outcomes among critically ill children.

**Methods:**

Diaphragmatic function was assessed by diaphragm ultrasound. According to the result of diaphragmatic ultrasound, all enrolled subjects were categorized into the DD group (*n* = 24) and the non-DD group (*n* = 46). Collection of sample characteristics in both groups include age, sex, height, weight, primary diagnosis, complications, laboratory findings, medications, ventilatory time and clinical outcomes.

**Results:**

The incidence of DD in this PICU was 34.3%. The level of CRP at discharge (*P* = 0.003) in the DD group was higher than the non-DD group, and duration of elevated C-reactive protein (CRP) (*P* < 0.001), sedative days (*P* = 0.008) and ventilatory treatment time (*P* < 0.001) in the DD group was significantly longer than the non-DD group. Ventilatory treatment time and duration of elevated CRP were independently risk factors associated with DD. Patients in the DD group had longer PICU length of stay, higher rate of weaning or extubation failure and higher mortality.

**Conclusion:**

DD is associated with poorer clinical outcomes in critically ill childern, which include a longer PICU length of stay, higher rate of weaning or extubation failure and a higher mortality. The ventilatory treatment time and duration of elevated CRP are main risk factors of DD in critically ill children.

**Trial registration:**

Current Controlled Trials ChiCTR1800020196, Registered 01 Dec 2018.

## Background

As the primary muscle for respiration, the diaphragm generates nearly three- to four-fifths of the inspiratory capacity [1]. A remarkably negative effect of diaphragmatic dysfunction or **DD** is exerted on the respiratory ability, a general complication in critically ill adult patients, particularly among those on mechanical ventilation (**MV**) [2]. The study conducted by Goligher et al. reveals that in about 50% of patients on MV, there is a rapid decrease in the thickness of the diaphragm after intubation [3]. The primary reason for DD is MV, with significant atrophy of the diaphragm in 18–69 h in absolutely controlled ventilation [4]. In children with a critical illness, MV is widely used as a conventional treatment process of pediatric intensive care unit (**PICU**); a significant number of children (30%) in the PICU were given the support of MV [5], and nearly two-fifth (38%) of them suffered from DD [6]. There is a high risk for difficult, delayed weaning and failed extubation, in DD patients, besides the increased possibility of poor functional outcomes, a longer stay in the PICU, and death [7–9].

There are several reports on the causes of DD and associated clinical outcomes in adult patients with a critical illness. However, there are not many studies on DD in pediatric patients with a critical illness, the associated risk factors, with limited information on its impact on clinical outcomes in children [10–12]. Therefore, there is little experience in clinical identification, proper intervention and prognostic improvement of children in such cases.

In this study, risk factors in relation to DD were identified, short-term clinical outcomes and the role of DD were assessed in patients below 18 years of age, and the suggestion of any possible association of DD with poor clinical outcomes was evaluated among seriously ill pediatric patients in the PICU.

## Methods

### Subjects

This study was carried out at the academic 57-bed PICU, First Hospital, Jilin University in China. Seventy consecutive patients (age less than 18 years) were enrolled with the requirement of invasive MV for more than one full day between January 2019 and January 2020. The hospital’s institutional ethics committee consented to the protocol followed in this study (ChiCTR1800020196). The guardians or parents of the enrolled children were informed of the protocol for which, they gave written consent and were also provided with an information sheet.

Each participant fulfilled the established criteria [13] for readiness for weaning (recovery in the primary disease cause, **PEEP** or positive end-expiratory pressure ≤ 5–10 cm H_2_O, PaO_2_/F_iO2_ > 200, F_iO2_ ≤ 50%, and hemodynamic stability when the vasopressors are lacking). The criteria for exclusion were known neuromuscular disease (like myasthenia gravis, Guillain-Barre, or amyotrophic lateral sclerosis), injury to the cervical spinal cord, pneumothorax, guardians or parents not willing to take part in the study.

### Study design

Ultrasound was performed on enrolled subjects to assess the diaphragm during the spontaneous breathing trial (**SBT**), carried out using support trials using a ventilator (Drager Evita 4) for half an hour at 5 cm H_2_O PEEP and 8 cm H_2_O pressure support. Five min post-SBT initiation, ultrasound measurements were taken. Each enrolled patient was either assigned to the DD- or the control (non-DD) group based on the outcome of diaphragmatic echo. DD was described as a **DTF** (diaphragmatic thickening fraction) of less than 20% at the time of tidal breathing [14]. Finally, the characteristics of patients and clinical outcomes of the two groups are compared.

### Evaluation of the diaphragm ultrasound

**Fig. 1:**
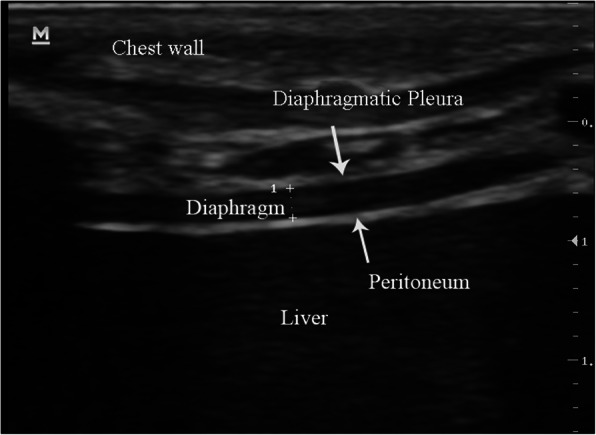
Ultrasound B-mode using a 10 MHz probe in the zone of apposition

The ultrasonography of the diaphragm was done by two experienced sonographers using a movable ultrasound apparatus from Mindray (M7 series, China) using a linear probe of 10HMz. The measurement of only the right hemidiaphragm was done because of better feasibility and repeatability of the right hemidiaphragm than the left hemidiaphragm [10]. The head of the bed was at an angle of 30-degrees and each subject was positioned semi-recumbently. Placement of the probe was done in the 8th to 11th intercostal space, between the antero-axillary or mid-axillary line, and perpendicular to the skin in a cranio-caudal direction to view the right hemidiaphragm properly [15]. The image analysis of the diaphragm ultrasound revealed a hypoechoic structure in the middle of two echoic lines (the peritoneal and the pleural membranes) (Fig. 1). In imaging of the B-mode, the estimation of **Tdi** (diaphragm thickness) was done from the internal edges of the pleural line to that of the peritoneal line at ends of both inspiration and expiration. DTF was calculated as (End-inspiration thickness – End-expiration thickness) / End-expiration thickness) [16]. In children, Tdi and **BW** (body weight) are correlated significantly [17]. Therefore, the standardization of Tdi was done by BW (DE/BW).

### Characteristics of patients and clinical outcomes

For each enrolled patient, the basic demographic were collected, in addition to the data on primary diagnosis, the levels of inflammatory factors at discharge and the period for which these inflammatory factors were elevated, and medications, because of the association of systemic inflammation with muscle atrophy in adult patients who were suffering from critical illness [18]. We observed the clinical outcomes including difficulty or delay in weaning, failure of extubation, duration on MV, duration of PICU stay and mortality.

### Analyses of statistical data

For clinical characteristics, demography, and DD and non-DD patient group outcomes, the comparison of continuous variables was done through the Mann-Whitney U test or Student t-test. Comparison of categorical variables was done through Fisher’s exact test or Chi-squared test. For continuous variables having normal distribution, presentation of data was done as mean ± standard deviation while that for variables with a non-normal distribution was done as median with interquartile range. For the description of categorical variables, n (%) was used. Logistic regression analysis was done to examine the factors significantly associated with DD, and we selected variables that remained statistically significant (*p* < 0.05) between DD and non-DD patient group. The SPSS Statistics, V22.0 from IBM Corp (Armonk, NY) for Windows was utilized for all analyses, and a *p*-value ≤0.05 was deemed significant statistically.

## Results

### Characteristics of samples

**Fig. 2:**
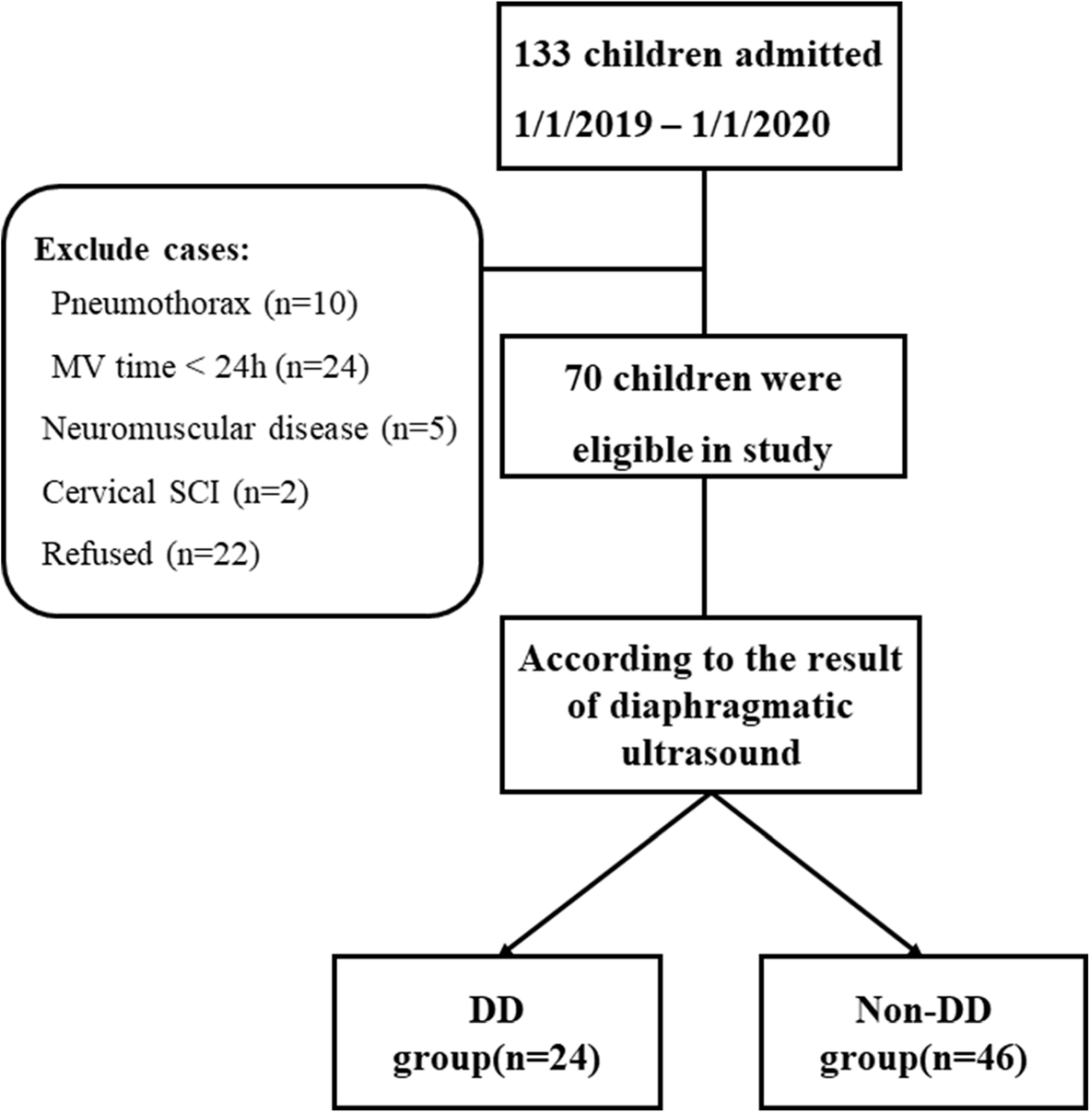
Flow chart of this study

In the period of study, enrolled children (*n* = 133) were given MV support. While 63 children were not included, eventually, 70 patients took part in this study. Those eligible were grouped either into the DD (*n* = 24) or non-DD (*n* = 46) groups as per the diaphragmatic echo result (Fig. 2).

A summary of the characteristics of all children is presented in Table 1. In this PICU, the DD incidence was 34.3% (24/70). On comparing the group characteristics, the following was revealed: the C-reactive protein **(CRP**) level at discharge was higher in the DD group compared to that in the non-DD group (IQR 4.50 [1.94–19.76] vs 1.81 [0.73–5.54], *P* = 0.003), and period of elevated C-reactive protein or CRP (IQR 18.50 [7.25–28.00] vs 4.50 [2.00–8.25], *P* < 0.001), days of sedation (IQR 8.00 [5.25–15.00] vs 6.00 [4.00–8.00], *P* = 0.008), and the ventilatory treatment time (IQR 13.00 [10.00–18.00] vs 5.50 [5.00–7.00], *P* < 0.001) were significantly more in the DD group than in the non-DD group. Nonetheless, no difference was observed between the two groups in the number of patients suffering from sepsis and treatment time of methylprednisolone, which are crucial factors in DD in adulthood.

**Table 1:** Sample characteristics

Characteristics	Non-DD Group(*n* = 46)	DD Group(*n* = 24)	*P*
Age, months, median (IQR)	33.50 (12.00–72.00)	45.50 (20.75–135.00)	0.25
Male sex (%)	67.39 (31/46)	58.33 (14/24)	0.45
Weight, kg, median (IQR)	14.90 (10.00–21.10)	17.75 (11.63–36.50)	0.23
Height, cm (mean ± SD)	95.00 (77.25–120.00)	100.00 (85.00–138.75)	0.23
BMI	16.95 (14.81–18.17)	16.60 (14.97–19.08)	0.75
Primary diagnosis, n (%)			
Respiratory dysfunction	70 (32/46)	67 (16/24)	
Cardiovascular dysfunction	2 (1/46)	4 (1/24)	
Other organ dysfunction	17 (8/46)	21 (5/24)	
Postoperative	11 (5/46)	8 (2/24)	
Sepsis	17.39 (8/46)	16.67 (4/24)	0.94
PCIS (mean ± SD)	81.37 ± 8.06	82.33 ± 6.03	0.61
Laboratory findings			
pH (mean ± SD)	7.47 ± 0.47	7.46 ± 0.57	0.93
PaO2, mmHg (mean ± SD)	95.23 ± 25.60	97.53 ± 26.54	0.73
PaCO2, mmHg median (IQR)	33.85 (30.30–37.35)	35.00 (32.00–42.75)	0.12
P/F median (IQR)	281 (231–356)	301 (239–430)	0.27
CRP at admission, μg/ml median (IQR)	18.87 (8.33–63.86)	14.52 (6.86–71.35)	0.70
CRP at discharge, μg/ml median (IQR)	1.81 (0.73–5.54)	4.50 (1.94–19.76)	**0.003**
Duration of elevated CRP, d median (IQR)	4.50 (2.00–8.25)	18.50 (7.25–28.00)	**< 0.001**
Sedative days, d, median (IQR)	6.00 (4.00–8.00)	8.00 (5.25–15.00)	**0.008**
Methylprednisolone, d, median (IQR)	5.00 (2.75–6.00)	5.00 (1.25–9.50)	0.57
Ventilation setting			
Mode	SIMV+PSV	SIMV+PSV	
PEEP, cmH_2_O	5	5	
Ventilatory treatment time d, median (IQR)	5.50 (5.00–7.00)	13.00 (10.00–18.00)	**< 0.001**
Diaphragm Indexs			
Tdi at end inspiration, cm/kg (IQR)	0.09 (0.06–0.13)	0.06 (0.05–0.13)	0.21
Tdi at end expiration, cm/kg (IQR)	0.06 (0.04–0.09)	0.06 (0.05–0.11)	0.77

***PCIS*** Pediatric Critical Illness Score, ***IQR*** interquartile range; ***SD*** Standard Deviation, ***BMI*** Body Mass Index, ***CRP*** C-Reactive Protein, ***PEEP*** Positive end-expiratory pressure

### DD development associated risk-factors and clinical outcomes

Table 2 presents the analysis results of the multivariable logistic regression. The risk factors independently associated with DD development included time for ventilatory treatment (95%CI, 1.18–3.38; OR, 1.99) and the period of elevated CRP (95%CI, 1.01–1.24; OR, 1.12).

**Table 2:** Multivariable logistic regression of risk factors associated with DD

Risk factor	OR	95% CI	*P*
Ventilatory treatment time, d	1.99	1.18-3.38	**0.010**
Sedative, d	0.76	0.54-1.07	0.12
Duration of elevated CRP, d	1.12	1.01-1.24	**0.039**
CRP at discharge, μg/ml	1.07	0.95-1.20	0.26

*OR* odds ratio, *DD* diaphragmatic dysfunction

The information in Table 3 reveals poorer clinical outcomes and a longer period of stay in PICU of the DD group patients (IQR 26.50 [15.00–35.50] vs 13.00 [10.00–18.00], *P* < 0.001), besides a higher failure rate of weaning (37.5% vs 10.87%, *P* = 0.008) or failed extubation (33.33% vs 8.7%, *P* = 0.009), a higher mortality in comparison with the non-DD group (20.83% vs 2.17%, *P* = 0.008).

**Table 3:** PICU clinical outcomes

Outcomes	Non-DD Group(n=46)	DD Group(n=24)	*P*
Delayed or difficult weaning (%)	10.87(5/46)	37.5(9/24)	0.008
Extubation failure (%)	8.70(4/46)	33.33(8/24)	0.009
Length of PICU stay, d, median (IQR)	13.00(10.00-18.00)	26.50(15.00-35.50)	<0.001
In hospital mortality (%)	2.17(1/46)	20.83(5/24)	0.008

*DD* diaphragmatic dysfunction, *PICU* pediatric intensive care unit

## Discussion

This study is the first, as per our knowledge, to evaluate factors related to the DD development and clinical outcomes in severely ill pediatric population. We observed a 34.3% DD incidence rate in children, as well as the association between the time for ventilatory treatment and the period of elevated CRP in the occurrence of DD. Thus, these outcomes implicate MV as not the exclusive cause of DD, with multiple factors playing a role in DD development in seriously ill children. For the first time in studies done to date, we report a relationship between elevated CRP duration and DD in children with a critical illness, indicating an important risk factor in the form of the body’s inflammatory response. We also found that pediatric patients with DD exhibit poorer outcomes, such as a longer period of stay in PICU, enhanced rates of extubation failure or weaning and increased mortality.

The outcomes of our analyses are in accordance with those obtained after assessing DD in hospitalized adult patients. In these patients, we observed a longer time of total ventilation, increased rates of failed extubation or weaning and mortality than in children who did not have DD [6, 19, 20]. Few reports on DD in severely ill children observed an independent association of respiratory weakness with longer time of ventilation and reintubation [9, 21]. In our previous study, we observed worsened diaphragmatic function in patients with failed weaning [22]. This suggests a close association of poorer clinical outcomes with DD in both children and adults. In mechanically ventilated adults, there was a 40–60% prevalence of dysfunction of the diaphragm diagnosed through ultrasound [8, 23, 24]. These values are higher than those observed in the current study. The primary cause for these may be, yet immaturely developed auxiliary inspiratory muscles in children; in adults, a greater role is played by the diaphragm [25]. Thus, a higher diaphragm baseline function is seen in children than in adults. The second cause may be that majority of adult patients suffered from chronic obstructive pulmonary disorder (**COPD**), where muscle fibers of the diaphragm undergo chronic oxidative remodeling, resulting in weakness in the ability of diaphragm compensation [26]. Third, the average time for ventilatory treatment was more the elderly than that in the children with DD [576 (374–850) hrs vs 360 (168–528)] [6, 22]. In addition, in critically ill children, we observed that the time of ventilation is a risk factor independent of DD, similar to that observed in adult studies [2, 27]. Further, the association of elevated CRP with DD development is another important observation in this study. In the diseases implicated in skeletal muscle dysfunction, an important contributor to the pathology is inflammation [28]; few studies on adults revealed that the risk factor with most significant impact for ICU-acquired weakness(**ICU-AW**) is systemic inflammation [18, 29]. Even post-critical illness, inflammation is prevalent and is not related to good recovery physically [30]. Nevertheless, the function of respiratory muscle imflammation remains unexplored largely, particularly in children with critical illnesses. In this study, we observed that the main risk factor of DD may be the period of the inflammatory response, although the rate of casualties cannot be estimated through our findings, and a randomized control trial (**RCT**) can demonstrate the same.

There were a few limitations to this study. First, the sample size was relatively small and may constrain the validity. The second limitation is the establishment of the criterion of ultrasound diagnosis (DTF < 20%) for DD from studies in adults and further study is needed in the pediatric population to ascertain the use of this reference value in children. Third, here, only CRP was used to represent patient inflammatory response, thus limiting the accuracy of our findings. Hence, more inflammatory factors like **IL-6** (interleukin 6), **IL-8** (interleukin 8), and **TGF** (transforming growth factor) must be included for a better indication of, to confirm our findings. Fourth, due to the small sample size in our study, a few crucial components in adult research like sepsis did not yield positive outcomes. While this does not directly associate sepsis with DD, the sample size must be expanded and the RCT must be conducted. Finally, the clinical outcomes of only the children with DD in the period of their stay at the hospital were investigated, and the patients post-discharge were not followed up. Dres et al. [4] showed that the frequency of DD is two times as that of weakness of the limb muscle, which associates significantly with post-discharge mediocre physical function [31–33]. Thus, the assessment of the effect of DD on the long-term prognosis of the pediatric population with critical illness is imperative.

## Conclusions

The clinical outcomes are remarkably affected by DD in children with critical illnesses, including a longer stay at the PICU, an enhanced rate of failed extubation and weaning, and a higher rate of mortality. The duration and time of ventilation of increased CRP are primary risk factors of DD development in children with critical illness. Particularly, an earlier DD identification in pediatric population with critical illnesses is vital to protect the functions of the diaphragm. However, research in children has been relatively inadequate, and more studies on the pediatric population with DD are needed for better respiratory and clinical rehabilitation.

## Data Availability

The datasets used and/or analyzed during the current study are available from the corresponding author on reasonable request.

## Abbreviations

PICU: Pediatric intensive care unit
MV: Mechanical ventilation
PCIS: Pediatric critical illness score
SBT: Spontaneous breathing test
Tdi: Diaphragm thickness
DTF: Diaphragmatic thickening fraction
IQR: Interquartile range
SD: Standard Deviation
PEEP: Positive end-expiratory pressure
COPD: Chronic obstructive pulmonary disease
BW: Body weight
CRP: C-reactive protein
DD: Diaphragmatic dysfunction
OR: Odds ratio
RCT: Randomized contral study
TGF: Transforming growth factor
IL-8: Interleukin 8
IL-6: Interleukin 6
ICU-AW: ICU-acquired weakness
